# Regular consumption of lacto-fermented vegetables has greater effects on the gut metabolome compared with the microbiome

**DOI:** 10.1017/gmb.2023.9

**Published:** 2023-06-29

**Authors:** Kylene Guse, Ashok Sharma, Emily Weyenberg, Sam Davison, Yiwei Ma, Yuni Choi, Abigail J. Johnson, Chi Chen, Andres Gomez

**Affiliations:** 1 Department of Food Science and Nutrition, University of Minnesota, St. Paul, MN, USA; 2 Department of Animal Science, University of Minnesota, St. Paul, MN, USA; 3 Division of Epidemiology and Community Health, School of Public Health, University of Minnesota, Twin Cities, Minneapolis, MN, USA

**Keywords:** fermented foods, gut microbiome, lacto-fermented vegetables, gut metabolome, short-chain fatty acids, dietary diversity

## Abstract

The industrialisation of Western food systems has reduced the regular consumption of lacto-fermented vegetables (LFV). Consuming LFV may exert health benefits through the alteration of the gut microbiome, but the mechanisms involved remain unclear. To start understanding the possible benefits of LFV, we compared faecal microbial diversity and composition, as well as dietary habits between individuals who regularly consume LFV (*n* = 23) and those who do not (*n* = 24). We utilised microbial DNA amplicon sequencing (16S rRNA and ITS2) and untargeted metabolomics (LC–MS) to analyse stool samples. Study participants also provided three consecutive days of dietary data. Results show minor effects on microbiome composition; with the enrichment of a few microorganisms potentially associated with vegetable ferments, such as *Leuconostoc mesenteroides and Rhodotorula mucilaginosa* (*P* < 0.05), in LFV consumers. However, LFV consumption had greater effects on the faecal metabolome, with higher abundances of butyrate, acetate, and valerate (*P* < 0.05) and significantly greater metabolome diversity (*P* < 0.001). Overall, the observations of minor changes in the faecal microbiome and greater effects on the faecal metabolome from LFV consumption warrant further investigations on the health significance of LFV as regular components of the daily diet in humans.

## Introduction

Fermentation of dietary staples is or has been a common practice in many cultures (Dunn et al., 2021). Archaeological evidence shows that humans have engaged in food fermentation since at least 4300 B.C. (Amato et al., 2021). However, other evidence suggests that evolutionary changes in human genes for metabolising ethanol and for interacting with lactic-acid bacteria (LAB) are accordant with a much earlier association with fermented foods, dating back to the emergence of hominids from other primates (Carrigan et al., 2014; Peters et al., 2019; Janiak et al., 2020; Amato et al., 2021). The transition from hunter-gatherer societies to more settled communities also required survival strategies in the form of food preservation, which made fermented foods staples of the human diet (Marco et al., 2020; Tamang et al., 2020). Thus, the human propensity for fermented food consumption is rooted in ancestral behaviours and survival strategies (Amato et al., 2021).

However, the adoption of industrialised food systems, characterised by highly sanitised environments and extremely processed, packaged foods, has, in general, become a major source of the daily diet in Western countries. One potential consequence of food industrialisation is that human populations have been deprived of diverse microbial exposures (Marco et al., 2020). In turn, food industrialisation has also coincided with low gut microbiome diversity, generating questions about how this loss has contributed to the higher incidence of metabolic and inflammatory diseases observed in the industrialised Western world, compared with populations that still rely on traditional diets for subsistence (Carrera-Bastos et al., 2011; Pontzer et al., 2012). To address this public health issue, diets targeting the gut microbiome to introduce or enhance certain taxa or specific functionalities have been proposed in precision nutrition and medicine (Wastyk et al., 2021).

Fermented foods have gained increasing attention, with some researchers supporting the addition of safe, live microbes in the daily diet to maintain health (Marco et al., 2020). While dairy-based fermented foods have been more widely studied and shown to have beneficial effects on health (Matsumoto and Benno, 2006; Wang et al., 2013; Buendia et al., 2018; Pimentel et al., 2018; van de Wouw et al., 2020), less is known on the specific benefits of LFVs such as sauerkraut and kimchi. Furthermore, despite a recent interest in studies focused on the interaction between different kinds of fermented foods and microorganisms in the human gut microbiome, the mechanisms by which these foods exert health benefits are not yet understood (Douillard and de Vos, 2019; Chan et al., 2021).

To shed more light on the impacts of LFV on the gut microbiome of Western humans, we collected dietary records and profiled faecal bacterial and fungal communities along with the metabolome in 47 faecal samples from two groups based on the regular consumption (consumers, n = 23) or no consumption (non-consumers, n = 24) of LFV. By investigating the diet, microbiome, and metabolome of regular consumers and non-consumers of LFV, we intend to contribute to further understanding of the possible mechanisms of action and health benefits of LFV.

## Methods

### Study population

Healthy adults between the ages of 20 and 50 were recruited from the Minneapolis/St. Paul metro area mainly through social media outlets such as Facebook and Instagram to participate in a study investigating how fermented foods impact the gut microbiome. Healthy participants were recruited as the “consumers” of lacto-fermented vegetables (LFV Consumers) if they consumed at least 1 serving (2 ounces), 5 times a week, and had been doing so for the last two years. Healthy participants were recruited as “non-consumers” if they reported never consuming lacto-fermented vegetables in the last two years. All participants were asked to limit any other fermented food intake, especially yogurt, two weeks prior to providing a stool sample.

### Ethics

This study was conducted according to all guidelines outlined in the Declaration of Helsinki, and all procedures involving human participants were approved by the University of Minnesota Institution Review Board (IRB – STUDY00004208). Written informed consent was obtained before subjects participated in any study procedures.

### Data collection

Upon enrollment in the study, all participants received a dietary record journal developed by a registered dietitian and a stool sample collection kit. Research team members instructed participants to record all food and drink intake for three consecutive days and to include one weekend day (example Thursday – Saturday), followed by instructions on how to take a stool sample on the fourth day. LFV consumers were asked to increase their fermented vegetable intake to at least 4 servings (4 ounces) of LFV every day for two weeks prior to recording dietary data and taking a stool sample. Participants collected their stool sample on a sterile faeces catcher and were instructed to collect 1 gram of stool into the faecal DNA/RNA shield collection tubes (Zymo Research, Irvine, CA). Upon collection of the dietary data and faecal sample, participants were instructed to contact research team members for pick-up. Stool samples were stored in −80 °C. Three research team members entered participant dietary record data into the Automated Self-Administered 24-hour Dietary Assessment Tool (ASA24), an open-source web-based software system designed to capture daily dietary intake, for dietary data analysis.

### Microbiome analyses

#### DNA extraction, sequencing, and data processing

Genomic DNA was extracted using the Power Soil DNA extraction kit of MoBio (Carlsbad, CA). To determine the bacterial composition, the V4 variable region of the 16S rRNA gene was amplified using 16S-515F (GTGCCAGCMGCCGCGGTAA) and 16S-806R (GGACTACHVGGGTWTCTAAT) primers. To examine the fungal composition, the internal transcribed spacer 2 (ITS2) by amplification of ITS3 (GCATCGATGAAGAACGCAGC) and ITS4 (TCCTCCGCTTATTGATATGC) primers were used. Sequencing of pooled libraries was carried out using the Illumina MiSeq platform at the University of Minnesota Genomics Center (UMGC) to generate 2*300 bp of sequences. 16S rRNA and ITS2 sequences were processed using custom-made Perl scripts and the Qiime2 pipeline (qiime2.org). Briefly, raw sequencing data were processed to remove primers and low-quality reads (phred quality score < 30). These high-quality reads were considered for denoising, merging, and chimera removal, and to generate unique amplicon sequence variants (ASVs) using the Dada2 plugin within Qiime2 (Bolyen et al., 2019). Representative sequences of each ASV were aligned using MAFFT, and phylogenetic trees – both rooted and unrooted – were constructed with FastTree (Price et al., 2009). Taxonomic assignments of bacterial ASVs were carried out by trained naive Bayes classifiers on reference sequences (clustered at 99% sequence identity) from Greengenes 13_8, and fungal ASVs were carried out with the UNITE database. For both taxonomic assignments, Qiime2 plugins feature-classifier fit-classifier naive Bayes and feature-classifier classifier-sklearn were used (Bolyen et al., 2019). The generated bacterial and fungal ASV count and frequency tables were converted to relative proportions using total reads per sample, and the ASVs which were not present in at least 5 samples (~3% of total samples) were omitted from the data set.

### Metabolome analyses

#### Sample preparation for LC–MS analysis

Stool samples were originally collected using a guanidinium-based solution, and extracted by vortexing and sonication for 10 min, and centrifuged at 18,000× g for 10 min to remove the insoluble fraction. All samples were stored at −80 °C.

#### Chemical derivatisation

For detecting organic acids, which are the main products of carbohydrate fermentation, the samples were derivatised with 2–2′-dipyridyl disulfide (DPDS), triphenylphosphine (TPP), and 2-hydrazinoquinoline (HQ) prior to the LC–MS analysis (Lu et al., 2013). Briefly, 2 μL of sample or standard was added into 100 μL of freshly prepared ACN solution containing 1 mM DPDS, 1 mM TPP, 1 mM HQ, and 100 μM deuterated *d_4_*-acetic acid as internal standard. The reaction mixture was incubated at 60 °C for 30 min, chilled on ice, and mixed with 100 μL of H_2_O. This mixture was centrifuged at 18,000 × g for 10 min, and the supernatant was transferred into an HPLC vial for LC–MS analysis.

#### LC–MS analysis

The workflow of LC–MS analysis was described previously by Ma et al. (2019). Briefly, 5 μL aliquot of diluted stool samples was injected into an Acquity ultra-performance liquid chromatography (UPLC) system (Waters, Milford, MA, USA) and separated in a BEH C18 column with a gradient of mobile phase ranging from water to 95% aqueous acetonitrile consisting of 0.05% acidic acid in a 10 min run. The LC eluate was directly introduced into a Xevo-G2-S QTOF mass spectrometer for accurate mass measurement and ion counting. Capillary voltage and cone voltage for electrospray ionisation (ESI) was maintained at 3 kV and 30 V for positive-mode detection, respectively. Nitrogen was used as both cone gas (50 L/h) and desolvation gas (600 L/h) and argon as collision gas. For accurate mass measurement, the mass spectrometer was calibrated with sodium formate solution (range *m/z* 50–1000) and monitored by the intermittent injection of the lock mass leucine encephalin ([M + H]^+^ = *m/z* 556.2771). Mass chromatograms and mass spectral data were acquired and processed by MassLynx™ software (Waters, Milford, MA, USA) in centroided format. Additional structural information was obtained by tandem MS (MS/MS) fragmentation with collision energies ranging from 15 to 45 eV.

#### Multivariate data analysis

Chromatographic and spectral data of samples were analysed using MarkerLynx software (Waters). A multivariate data matrix containing information on sample identity, ion identity (retention time (RT) and *m/z*), and ion abundance was generated through centroiding, deisotoping, filtering, peak recognition, and integration. The intensity of each ion was calculated by normalising the single ion counts (SIC) versus the total ion counts (TIC) in the whole chromatogram. The data matrix and sample list were further exported into SIMCA-P^+^ software (Umetrics, Kinnelon, NJ, USA). The chemical identities of metabolite markers were determined by elemental composition analysis; database search Metlin (accessed on 16 January 2021), ECMDB (accessed on 16 January 2021), HMDB (accessed on 16 January 2021), and KEGG (accessed on 16 January 2021); fragmentation; and comparison with authentic standards if possible.

### Statistical analyses

#### Dietary analysis

Three members of the research team entered dietary records into the Automated Self-Administered 24-hour (ASA24) Dietary Assessment Tool, version (2018), developed by the National Cancer Institute, Bethesda, MD (https://epi.grants.cancer.gov/asa24). ASA24 determines the nutritional composition of foods and assigns nutrient information to them using the USDA’s Food and Nutrient Dietary Database (FNDDS 2013–2014). Although sauerkraut and kimchi were available in the nutrient database, some LFV consumers ate other vegetable ferments such as fermented carrots, fermented chilis, and fermented peppers. Since we were interested in fermented vegetable intake quantity, and these ferments were not available in the database, these fermented foods were entered into ASA24 as sauerkraut. Based upon the analytic files provided by ASA24, we calculated an average of 65 nutrients and 37 food groups. We then calculated the Healthy Eating Index Scores. The HEI-2015 total scores (theoretical range = 0–100) were estimated based on established National Cancer Institute procedures and SAS code from 13 components of dietary intake (*Steps for Calculating HEI Scores*, 2015): total fruits, whole fruits, total vegetables, dark green vegetables and legumes, whole grains, total dairy, total protein foods, seafood and plant proteins, refined grains, added sugars, fatty acids, sodium, and total saturated fats. HEI scores are based on density values or ratios of intake per total energy. The resulting ratios are compared with the applicable standards for scoring (*Steps for Calculating HEI Scores*, 2015). Standards for HEI-2015 are shown in Supplementary Table S1. A Shapiro–Wilkes normality test determined total dairy and refined grains to be normally distributed and therefore, a Student’s T-Test was used to determine statistical significance for those categories, while a Wilcoxon rank-sum test was used to determine statistical significance for all other food component categories. To determine the LFV source, a manual review of all consumers’ dietary records was completed and labelled as homemade (if the ferment was made at home), local (if the ferment was made by a small producer in the Midwest), and national (if the ferment was made by a company outside the Midwest).

#### Dietary food tree analysis

Since dietary patterns have a strong influence on human health, and previous studies have found that, similar to the microbiome, dietary intake is personalised, we took an ecological approach to the dietary record analysis (Johnson et al., 2019). The diversity methods used for microbiome data analysis were applied to the dietary data diversity analysis. We applied a phenetic, hierarchical tree of foods from the Food and Nutrient Database for Dietary Studies (FNDDS) (U.S. Department of Agriculture and Agricultural Research Service 2017–2018) developed by Johnson et al. (2019), which allowed us to apply a UniFrac, tree-based beta diversity metric that provides statistical information across related foods to measure the overall diversity of total foods consumed by LFV consumers compared to the non-consumers. Food trees were calculated from FoodTree output using Qiime2 2020.6 (Bolyen et al., 2019). The dehydrated gram weight of consumed foods and drinks was calculated using the individual foods file from ASA24 by subtracting the moisture content from the gram weight of each food. An alpha diversity metric (Shannon diversity index) was applied in the R statistical platform utilising the vegan package (Oksanen et al., 2020), and a Wilcoxon rank-sum test was used to determine statistical significance between LFV consumers and non-consumers.

#### Microbial community analysis

Microbial community analyses were performed in the R statistical platform. Briefly, for alpha diversity, beta diversity, and permutational multivariate analyses of variance (PERMANOVA), multiple R packages such as vegan, ape, phyloseq were used (Paradis et al., 2004; Oksanen et al., 2007; McMurdie and Holmes, 2013). Inter-individual variation or intra-group dispersion was calculated using betadiver/betadisper functions in the R vegan package. Bacterial and fungal differential abundance analyses were visualised in volcano plots within the Metaboanalyst 5.0 online platform (Pang et al., 2021). Wilcoxon rank-sum tests were used to assess the statistical significance of differential gut microbes between groups. Co-occurrence probability networks were designed to show microbe–metabolite interactions using Microbe–Metabolite vectors (MMVEC) within Qiime2 2020.6, and graphical network visualisation was conducted in Cytoscape 3.8.2. We considered only the highest positive conditionals (conditionals ≥4.0) and created networks based upon the degree of connectivity (node size) and the strength of probability between metabolites and bacterial ASVs (thickness of edges).

#### Metabolomic analysis

Metabolomic analyses were conducted within the Metaboanalyst 5.0 platform (Pang et al., 2021). Data were normalised by sum, log-transformed, and auto-scaled (mean-centered divided by the square root of the standard deviation of each variable). Then, differential abundance analysis (volcano plots based on FDR-adjusted, Wilcoxon rank-sum tests) and partial least square discriminant analysis (PLS-DA) were conducted and cross-validated by 1,000 permutations. In addition, univariate analyses were performed on the discriminant metabolites found for each group, as well as metabolites identified as short-chain fatty acids (valeric acid, butyric acid, acetic acid, propionic acid). To test statistical significance, Wilcoxon rank-sum tests were used using the wilcox.test function in R.

## Results

A total of 29 LFV consumers and 27 non-consumers were recruited for the study. However, 23 LFV consumers and 24 non-consumers met all the inclusion/exclusion criteria and completed the study (Supplementary Figure S1 and Supplementary Table S2). The majority of LFV consumer participants were white (82.6%), female (82.6%), and an average age of 33. A majority of non-consumer participants were white (87.5%), female (58.3%), and an average age of 29. Two non-consumer participants did not provide dietary data. For a detailed list of participant demographics and average LFV intake, please see Supplementary Tables S3 and S4.

### Consumers of fermented vegetables showed minor differences in background diet and lifestyle behaviours compared to non-consumers

**Figure 1.**
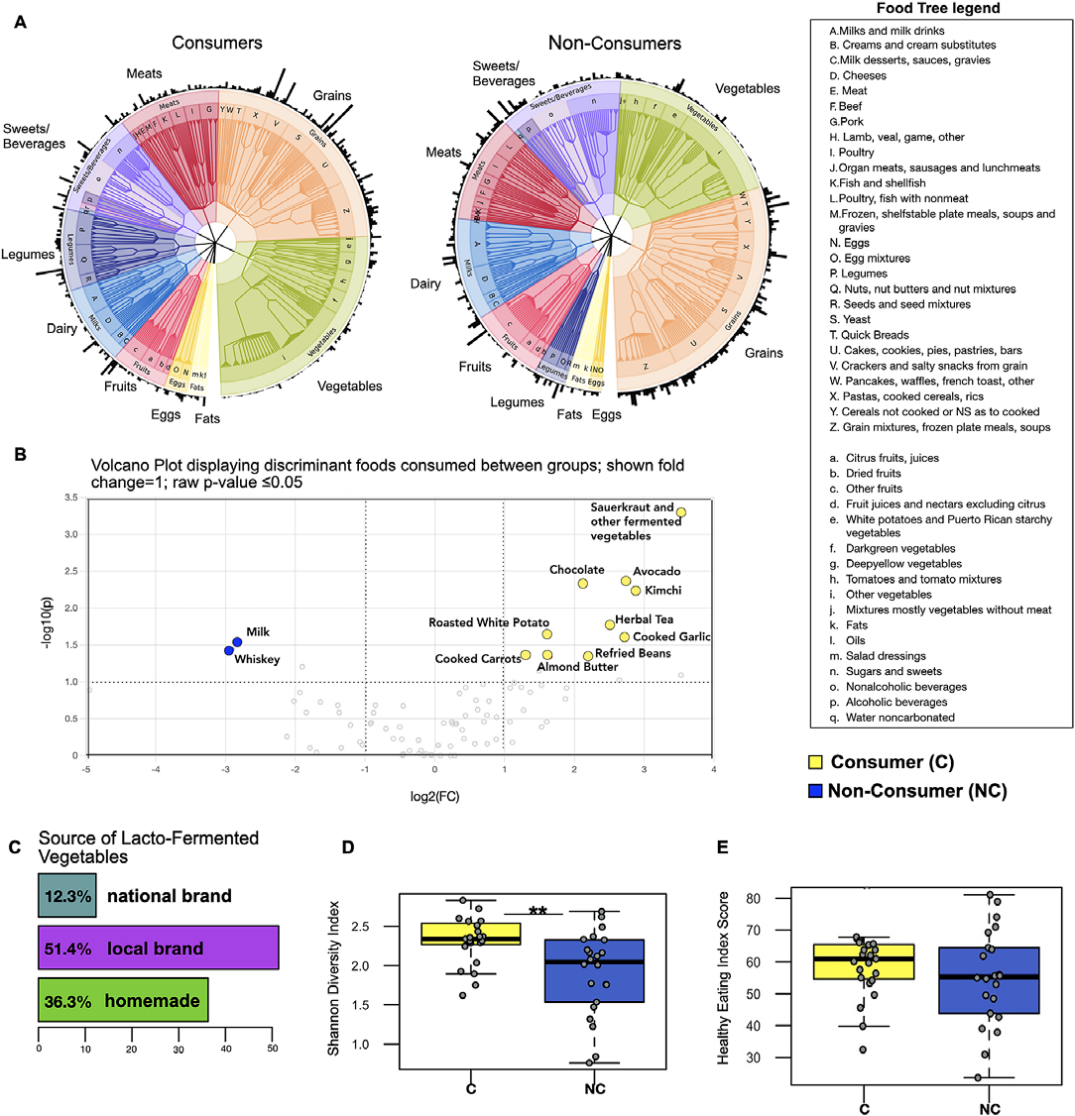
LFV consumers display only minor differences in background diet compared to non-consumers. (A) The food trees are phenetic representations of dietary groups for multivariate analysis of 24-hour food records across three consecutive days. Outer edges represent the relative abundance of each food. (B) Volcano plot showing results from differential abundance analysis in dietary composition between consumers and non-consumers – X axis shows log2 fold changes (FC) and Y axis shows -log10 *P*-value (−log10 > 1 represents *P* < 0.05). (C) The majority of fermented vegetables consumed came from locally produced sources or were homemade. (D) Alpha diversity analysis showing higher food diversity in fermented vegetable consumers – ** indicates *P*-value ≤ 0.01 (E) Healthy Eating Index Score (59 out of 100 for LFV consumers and 55 out of 100 for non-consumers (HEI-2015).

A food tree revealed minor differences between consumers and non-consumers, with more emphasis on vegetable and legume intake and less sugar and grain intake observed in the former (Figure 1A). However, as expected, consumers exhibited frequent consumption of fermented vegetables (kimchi, sauerkraut, and other fermented vegetables, coded as sauerkraut in this analysis) as well as increased consumption of plant-based foods such as raw avocado, cooked carrots, cooked garlic, and almond butter (*P*-value ≤ 0.05; fold change =1; Figure 1B). Interestingly, foods found to be more frequently consumed by non-consumers were milk and whiskey (*P*-value ≤ 0.05; fold change = 1). We also analysed sources of LFV consumption and found that the majority of LFV consumed came from locally produced brands or were homemade (Figure 1C). In addition, we applied the Shannon diversity index to understand the overall diversity of foods consumed and found that LFV consumers demonstrated higher dietary diversity (Wilcoxon rank-sum test; *P* = 0.005; Figure 1D) despite the similarity in overall energy consumption (kcal) between the two groups (Supplementary Figure S2).

Taken together, the dietary intake analyses revealed a somewhat healthier eating pattern in the consumers. Therefore, we calculated the average intake over the three days of dietary records for total vegetables, grains, legumes, and sugar, as well as the Healthy Eating Index (HEI -2015) to test the overall dietary pattern quality between the two groups. While consumers tended to emphasise more on vegetable and legume intake and slightly less on grain, sugar, and dairy intake, results did not show statistical significance (Supplementary Figure S2). The HEI analysis indicated a mean score of 59 for the consumers out of 100, which is slightly higher than the mean score of the non-consumers (55), but there was not a significant difference between groups (HEI 2015; Wilcoxon rank-sum test; *P*-value = 0.13; Figure 1E). These scores mirror the HEI-2015 national average for Americans, which is 58 out of 100. The two groups did not differ in their dietary macronutrients (Supplementary Figure S2). However, they did differ in some of the HEI-2015 Food Component categories. Non-consumers demonstrate more significant Dairy intake, while LFV consumers show more Total Protein and Seafood and Plant Protein intake (Supplementary Figure S3 HEI 2015 components).

### LFV consumers and non-consumers show minor differences in faecal bacterial community composition

**Figure 2.**
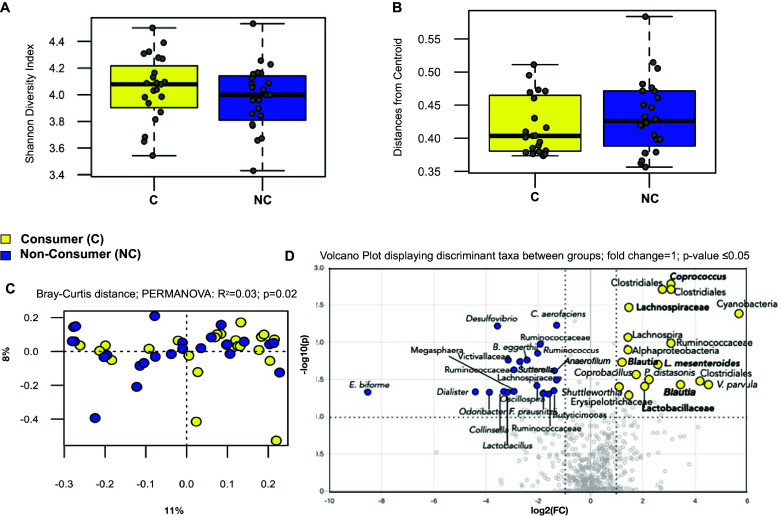
Gut bacteriome shows minor differences between LFV consumers compared to non-consumers (A) Alpha diversity analysis shows no significant difference between groups. (B) Distances from centroid show no differences in inter-individual variation in bacterial composition. (C) Weighted Bray–Curtis distances between the consumers and non-consumers. (D) Volcano plot reveals microbes commonly found in LFV such as *Leuconostoc mesenteroides* – X axis shows log2 fold changes (FC) and Y axis shows – log10 *P*-value (−log10 > 1 represents *P* < 0.05).

No statistically significant differences between consumers and non-consumers were observed in bacterial alpha diversity metrics (Shannon’s diversity index, Wilcoxon rank-sum test; *P* = 0.35; Figure 2A and Faith’s phylogenetic diversity; Wilcoxon rank-sum test; *P* = 0.44; Supplementary Figure S4). Inter-individual variation (or group dispersion) patterns between the two groups did not display any differences (Wilcoxon rank-sum; *P* = 0.42; Figure 2B). Beta diversity measured by an unweighted Bray–Curtis, as well as weighted and unweighted UniFrac did not show any statistical significance between the two groups (Supplementary Figure S4). A weighted Bray–Curtis distance ordination analysis displays minor but significant stratification of the faecal bacteriome between consumers and non-consumers (PERMANOVA; R2 = 0.03; pseudo*-F-*statistic = 1.5; *P*-value = 0.02; Figure 2C). Volcano plots (fold change =1; *P*-value ≤ 0.05) showed that consumers exhibited an increased relative abundance of some lactic-acid bacteria commonly found in LFV such as *Leuconostoc mesenteroides,* among other taxa (Figure 2D and Supplementary Figure S5).

### Fungal community composition is not significantly different between LFV consumers

**Figure 3.**
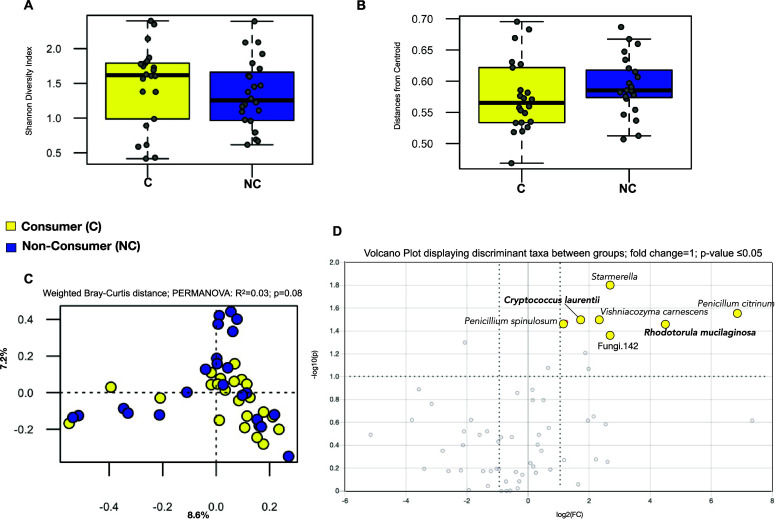
Gut mycobiome composition between groups (A) Alpha diversity analysis shows no significant difference between groups. (B) Distances from centroid show no differences in inter-individual variation in fungal composition. (C) Weighted Bray–Curtis distances show no significance between the consumers and non-consumers. (D) Volcano plot reveals fungal genera and species found in the consumers of LFV – X axis shows log2 fold changes (FC) and Y axis shows -log10 *P*-value (−log10 > 1 represents *P* < 0.05). No fungi were found to differentiate the non-consumers.

Our results indicate no difference in fungal alpha diversity between consumers and non-consumers (Shannon’s diversity index, Wilcoxon rank-sum test; *P*-value = 0.28; Figure 3A and Faith’s phylogenetic diversity; Wilcoxon rank-sum test; *P*-value = 0.18; Supplementary Figure S6). Like the gut bacteriome, inter-individual variation in fungal communities did not show significant differences between the two groups (Wilcoxon rank-sum test; *P* = 0.15; Figure 3B). Weighted Bray–Curtis (Figure 3C) distance ordination did not reveal any significant differences in faecal mycobiome (PERMANOVA, R2 = 0.03, *P*-value = 0.08) nor did an unweighted Bray–Curtis or UniFrac distance ordination (Supplementary Figure S6). However, a differential abundance analysis revealed that only LFV consumers had distinguishing fungal species, including higher abundances of *Rhodotorula mucilaginosa*, *Penicillium*, *Starmerella*, *Cryptococcus laurentii*, and *Vishniacozyma carnescens* (fold change =1; *P*-value ≤ 0.05; Figure 3D). Boxplots displaying the abundance distribution of fungal species between groups can be seen in Supplementary Figure S7.

### LFV consumers display a significantly distinct faecal metabolome

**Figure 4.**
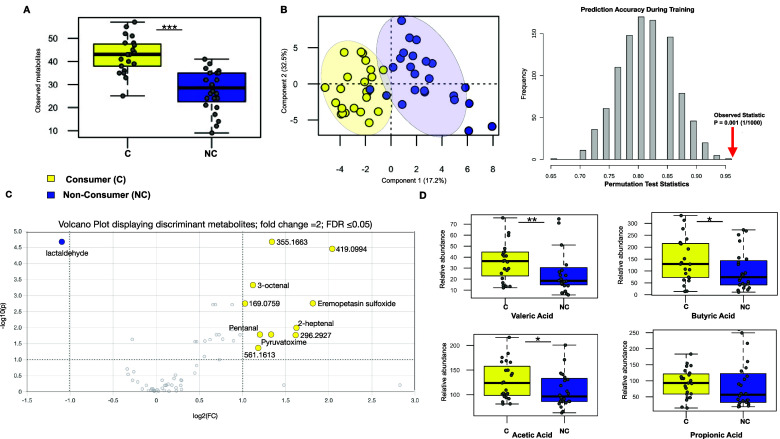
LFV consumers display a distinct faecal metabolome. (A) Box plots show high faecal metabolome diversity in the consumers. (B) PLS-DA model shows distinct separation in the faecal metabolome between the two groups, confirmed by permutation test statistic. (C) Volcano plot reveals discriminant faecal metabolites found the consumers and non-consumers of fermented vegetables – X axis shows log2 fold changes (FC) and Y axis shows -log10 *P*-value (−log10 > 1 represents FDR ≤ 0.05). (D) Box plots show relative abundances of short-chain fatty acids – valeric acid, butyric acid, and acetic acid, to be higher in the consumers, while propionic acid showed no statistical significance between the two groups: ** indicates *P*-value ≤ 0.01; * indicates *P*-value ≤ 0.05.

Contrary to the observation of microbial composition, we report significantly greater differences in the faecal metabolome between consumers and non-consumers. A power calculation demonstrating the significant discriminatory power of the metabolomics data (~0.84; n = 24; FDR = 0.1) has been provided in Supplementary Figure S8. Consumers displayed more richness in their faecal metabolite pool (Wilcoxon rank-sum test; *P* = 9.468e-07, Figure 4A). In addition, a partial least squares-discriminant analysis (Figure 4B) showed a clear distinction in the faecal metabolome (*P* = 0.001, Q2 = 0.74, R2 = 0.68), as well as a principal component analysis (PCA), which confirms clear differences between consumers and non-consumers (Supplementary Figure S9). More discriminant metabolites were found distinguishing the consumers (volcano plot, fold change = 2; FDR ≤ 0.05; Figure 4C); these include the metabolites tentatively identified as pentanal, 2-heptenal, and octenal through accurate mass-based database search, and several metabolites with unknown identities. For example, (ion 419. 0994^+^) was the most enriched signal in the faecal metabolome of consumers (Wilcoxon rank-sum test; *P* = 1.922e-06, Supplementary Figure S10). Lactaldehyde was found to be a discriminating metabolite among the non-consumers (Wilcoxon rank-sum test: *P* = 6.102e-07, S9, Supplementary Figure S10). In addition, the relative abundance of valeric, butyric, and acetic acids was found to be higher among consumers (one-tailed Wilcoxon rank-sum test; *P* = 0.02; *P* = 0.03, *P* = 0.02, respectively; Figure 4D).

### LFV consumers demonstrate different microbe–metabolite co-occurrence probabilities compared to non-consumers

**Figure 5.**
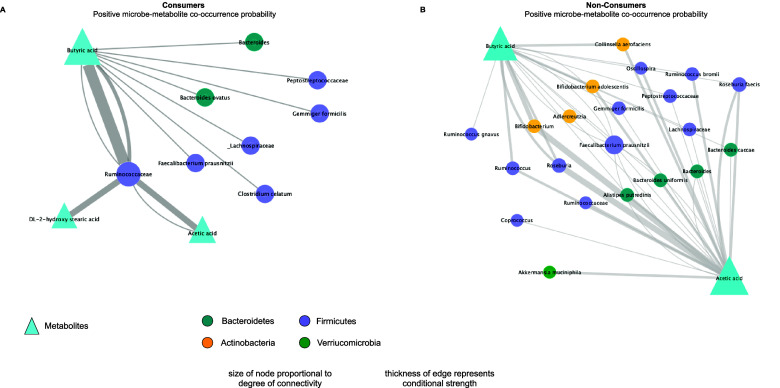
Co-occurrence probability networks between bacterial taxa and metabolites found in the LFV consumers and non-consumers. MMVEC analysis considering only the highest positive conditionals found (4.0–4.43). (A) Consumers demonstrate stronger but fewer interactions with butyric acid, while (B) non-consumers demonstrate more interactions with butyric acid and acetic acid, but strongest interactions within the latter. Color code in the shape of the node represents bacterial phylum. Edge thickness represents conditional or interaction (co-abundance) strength; node size represents degree of connectivity.

An MMVEC analysis displayed co-occurrence probabilities between microbial taxa and metabolites. We considered only the highest positive conditionals (conditionals ≥4.0) and created networks based upon degree of connectivity (node size) and the strength of probability between metabolites and bacterial ASVs (thickness of edges) (Figure 5A). Interestingly, the consumers demonstrated high co-occurrence probabilities of butyrate to a smaller number of microbes, mainly those belonging to the Firmicutes phylum. Ruminococcaceae is shown to have the highest co-occurrence probability with butyrate, acetic acid, and 2-DL-hydroxy stearic acid among consumers. Non-consumers display a much different network, showing higher diversity of microbes co-occurring with butyrate and more taxa associated with acetic acid (Figure 5B). No significant correspondence was found between fungal taxa and any metabolite.

## Discussion

Overall, these data show only minor effects on microbial community composition based on LFV consumption, but greater effects on their faecal metabolome. We report the enrichment of some bacteria and fungi typically associated with fermented vegetables and a richer and unique metabolite pool in regular LFV consumers. These distinguishing features, despite similar baseline diets between groups, may indicate the consumption of LFV triggers changes in the gut environment. Here, we discuss the potential significance of these results in the context of regular consumption of fermented vegetables.

### Dietary data shows similar baseline diets between LFV consumers and non-consumers but higher dietary diversity in the former

We considered the possibility that regular LFV consumers may be inclined to eat an overall healthier diet, which could bias microbiome and metabolome results. However, analyses of dietary data demonstrate a similar baseline diet and HEI score between the two groups. Although LFV consumers also demonstrate higher dietary protein foods including Total Protein Foods and Seafood and Plant Proteins categorised by the HEI-2015, it is difficult to assess the extent to which baseline diets may have impacted microbiome and metabolome results, apart from LFV consumption.

We observed a higher number of diverse foods (dietary diversity) besides the addition of LFV in the consumer group, which displayed an emphasis on plant-based foods such as raw avocado, cooked carrots, cooked garlic, and almond butter (Figure 2B). This may have impacted the microbiome and metabolome results presented here. Eating a wide variety of foods or increasing dietary diversity has been associated with an increase in gut microbiome and metabolome diversity (Heiman and Greenway, 2016; Xiao et al., 2022). Overall, a diverse number of whole and healthy foods has been promoted as part of a healthy diet (Kennedy, 2004). Yet, some observational studies indicate that dietary diversity depends on overall healthy eating patterns and consumption of individual nutrients (Lee-Sarwar and Ramirez, 2022). In this study, we found that higher dietary diversity coincided with higher metabolite diversity in the LFV consumers. Further investigations should focus on the mechanisms by which dietary diversity may increase metabolome diversity under LFV consumption and, importantly, whether the higher metabolome diversity, as reported here, leads to positive health outcomes.

### Consumers’ bacteriome and mycobiome show minor features of LFV consumption

Minor effects on gut microbiome composition upon fermented food consumption, as reported here, have been shown in two other recent studies (Taylor et al., 2020; Wastyk et al., 2021). However, the enrichment of *L. mesenteroides* in some LFV consumers is notable in this dataset (7 out of 23 participants in the LFV consumers). Strains of *L.mesenteroides* have been found to be major lactic-acid bacteria in the fermentation of kimchi and sauerkraut and are widely used starter cultures, especially in sauerkraut (Eom et al., 2008; Chun et al., 2017; Yi et al., 2017). *L. mesenteroides* has been cited for their probiotic properties in several studies (Eom et al., 2008; Chun et al., 2017; Yi et al., 2017). Furthermore, the viability of LAB in the distal gut from the stock of fermented vegetables consumed is dependent upon storage time, tolerance to low pH, bile salts, salivary enzymes, gastric juices, and adaptation to body temperature (Czerucka et al., 2007; Menezes et al., 2020; Chan et al., 2021). Thus, as *L. mesenteroides* was seen in 7 out of 23 consumers, its presence in consumers’ faeces needs to be evaluated relative to the time of consumption, but also in the context of how successfully it can reach the distal colon across different individuals and populations. Regardless, the detection of this potentially probiotic strain in this study motivates further research to evaluate how LFVs can be successful vectors of beneficial gut bacteria. Recent feasibility studies showed that, indeed, dietary interventions designed for the regular consumption of LFV are feasible and can change gut microbiome composition (Galena et al., 2022; Thriene et al., 2022).

It is also worth noting that in the LFV consumers, some of the most discriminant taxa are in the Firmicutes phylum, including *Coprococcus* and the Lachnospiraceae family. These results are similar to those found by Wastyk et al. (2021), reporting that increased fermented food consumption over time resulted in an increased abundance of members of the Lachnospiraceae family. Other studies have also observed an increase in the ratio of Firmicutes over other taxa after colonic fermentation of fermented foods (Chen et al., 2020; Chan et al., 2021). However, subjects in the Wastyk et al. (2021) study also consumed dairy ferments, which precludes the association of Lachnospiraceae exclusively with LFV consumption in this study. Regardless, many members of *Coprococcus* and Lachnospiraceae are known butyrate producers (Vital et al., 2014), which could be associated with the increase in butyrate abundances observed in consumers’ faeces.

Our results found no significant difference in fungal communities between groups, except for the differential abundance of some fungi in some consumers only, including *R. mucilaginosa.* This oleaginous microorganism and common environmental yeast has been identified in a number of indigenous fermented foods including cocoa, kefir (Menezes et al., 2020), Chinese rice wine (Wu et al., 2013), other fermented milks, and sauerkraut (Satora et al., 2020). Along these lines, we recently showed that faecal fungal communities across different primate species are highly influenced by diet and environment (Sharma et al., 2022), which is in accordance with previous studies showing the strong influence of diet on the mycobiome of mice and non-human primates (Sun et al., 2021). Thus, the mycobiome patterns observed herein, along with the fact that discriminant fungi were only found in consumers, may show that LFV could be a potential source of fungal seeding in the gut, likely impacting bacteriome–mycobiome gut composition. However, whether the fungi detected in these consumers are specifically linked to LFV consumption remains unknown.

### LFV consumers have a different and diverse faecal metabolome with higher abundances of SCFAs

One of the most notable findings reported herein is the significantly higher faecal metabolite richness detected in LFV consumers. Thus, an important question focuses on investigating if LFV consumption contributes to enriching the biochemical pool in the gut, which would potentially impact the gut microbiome and systemic health (Wilmanski et al., 2019). It has been suggested that dietary diversity may provide more substrates for the production of diverse metabolites by the gut microbiome, including those that interact with the host (Heiman and Greenway, 2016; Annunziata et al., 2020). Likewise, evidence suggests that consuming diverse foods, and how those foods are prepared, shapes the composition of the gut microbiome (Carmody et al., 2019).

Although we did not find a statistical difference in fiber intake between LFV consumers and non-consumers, we found the relative abundances of valeric acid, butyric acid, and acetic acid to be higher in the former. SCFAs are the most abundant product of bacterial fermentation of undigested fibers in the gut, and it has been well established that the gut microbiome affects host physiology through SCFA production (Morrison and Preston, 2016; Nagpal et al., 2018; Markowiak-Kopeć and Śliżewska, 2020; Skrzypecki et al., 2020; Zhang et al., 2021). Research has shown that a decrease in the abundance of specific SCFAs is associated with inflammatory bowel disease (IBD), metabolic diseases, obesity, autoimmune disorders, and even impaired brain function (Markowiak-Kopeć and Śliżewska, 2020; Silva et al., 2020; Zhang et al., 2021).

Of particular interest is the higher relative abundances of valeric, butyric, and acetic acid in LFV consumers. However, the mechanisms by which fermented foods influenced SCFA synthesis in this study are not well understood. It has been shown that probiotic microorganisms are effective in increasing the production of SCFAs (Nagpal et al., 2018; Liang et al., 2019; Markowiak-Kopeć and Śliżewska, 2020; Traisaeng et al., 2020). For example, a strain of *L. mesenteroides* (*L. mesenteroides* EH-1) isolated from a fermented food was shown to produce butyric acid from fermentation *in vitro* and *in vivo*, which activated free fatty acid receptor 2 (FFAR2) and reduced blood glucose levels as well as the pro-inflammatory cytokine IL-6 in a type 1 diabetic mouse model (Traisaeng et al., 2020).

Our microbe–metabolite network analysis shows there is a slightly higher abundance of butyric acid with a smaller number of microbes in LFV consumers, while the network in the non-consumers shows a much higher number of microbes co-occurring with a smaller abundance of butyric acid and a much higher abundance of acetic acid. Thus, since lactic acid was not detected in any samples in the faecal metabolome of LFV consumers, one possibility is that the high amounts of lactic acid found in fermented vegetables may be completely converted to SCFAs, particularly butyrate, by only a few taxa *or* that LFV consumption may promote the conversion of more SCFAs but only by certain species.

Likewise, evidence suggests that SCFAs and other metabolites present in fermented foods may be just as important as the microorganisms when it comes to exerting benefits on human health. These metabolites have been shown to activate G-protein coupled receptors (GPCRs), which are expressed in a large number of cells in the gastrointestinal, immune, and nervous systems (Silva et al., 2020). Dietary SCFAs are almost completely absorbed in the small intestine (Chambers et al., 2015). However, consumption of higher levels, such as those found in LFV, may increase levels in the plasma, which have been found to activate free fatty acid receptor 3 (FFAR3) and improve hepatic metabolic conditions in a high-fat diet–induced obese mouse model (Shimizu et al., 2019).

Furthermore, Peters et al. (2019) found that D-phenyllactic acid (D-PLA), a metabolite produced by lactic-acid bacteria and found in higher amounts in sauerkraut, is a potent activator of a GPCR for hydroxycarboxylic acid receptor 3 (HCAR3), which is a regulator of immune function and energy homeostasis only present in humans and other hominids but no other mammals (Peters et al., 2019). In an evolutionary context, this research suggests that the changing availability of food in certain ecological niches where hominids evolved could have selected for specific HCAR3 functions, such as a response to certain lactic-acid bacteria and their by-products (like D-PLA) (Peters et al., 2019). Taken together, this suggests that metabolites, both those produced by microbial fermentation in the gut *and* those obtained through the diet (such as those in LFVs), may activate critical GPCRs required for physiological homeostasis. As such, microbial-derived metabolites, both consumed in the diet and produced in the gut, may be one potential mechanism by which fermented foods exert benefits on human health.

### Study limitations

The small sample size is one of the main limitations of this study as it was difficult to recruit participants in the Minneapolis/St.Paul metro area that consumed LFV regularly and met all inclusion/exclusion criteria. Furthermore, the convenience sample of LFV consumers and non-consumers, including demographic representativeness (majority white female participants), may also impart bias. In addition, it should be noted that self-reporting of dietary data and the entry of these data into ASA24 by researchers could impart bias and error. The metabolomic analysis in this study was mainly conducted for examining any organic acids we could detect related to the consumption of fermented vegetables (for example, lactic acid and SCFAs). The samples were collected using the buffer (guanidinium-based) for microbiome analysis, and the LC–MS analysis was based on the HQ derivatisation reaction that specifically targets organic acids. This procedure limits the coverage of our metabolomic analysis and presents technical challenges in the characterisation of unknown metabolite markers, such as the ones in Figure 4C (due to the presence of both derivatised and underivatised signals in our data). Finally, this study was associative in nature and built upon pre-established LFV consumption patterns. This precluded us to make inferences on how fixed amounts of LFV consumed were associated with specific microbiome and metabolome patterns observed. We also do not present any evidence of specific host physiological functions, beyond the faecal microbiome and metabolome. As such, it is unknown if increased LFV consumption impacts health in this study.

## Conclusion

The resurgence of fermented foods in Western countries has prompted a surge in fermented food products, which have been categorised as healthy, “probiotic,” and functional foods. However, evidence of their purported benefits, particularly from specific kinds of fermented foods such as LFV and their possible impact on the human gut microbiome, is still insufficient. These results demonstrate that consumption of LFVs has minor impacts on gut microbial communities, except for a potential increase in butyrate producers and the probable presence of some ferment-derived microbes in some consumers (e.g., *L. mesenteroides and R. mucilaginosa).* However, the results point to more pronounced effects on the gut metabolome, including a more diverse faecal metabolite pool, as well as higher relative abundances of SCFAs.

Further research should determine how dietary diversity and/or LFV consumption can contribute to a specific metabolome environment in consumers. In addition, the mechanism involved in the greater generation of SCFAs in consumers should be explored in detail, considering both the role of ferment-derived microbes and the metabolites consumed along with the food. To that end, both controlled feeding (in vivo) and in-vitro trials are needed to determine how overall microbial and metabolite complexity in specific fermented foods like LFV can specifically drive microbes and metabolites across all gut segments (not just the distal colon), relative to the time of ingestion and consumer’s characteristics (different diets, environment, and genetic factors). Importantly, the potential of LFV consumption on specific markers of gut health (gut physiology and function) needs to be evaluated.

## Data Availability

All data is available by contacting the corresponding author.

## Supplementary material

For supplementary material accompanying this paper visit https://doi.org/10.1017/gmb.2023.9. click here to view supplementary material
