# Supplementary material for: Regular consumption of lacto-fermented vegetables has greater effects on the gut metabolome compared with the microbiome
Source: Gut Microbiome (Camb). 2023 Jun 29;4:e11. doi: 10.1017/gmb.2023.9 (PMC11406409; doi:10.1017/gmb.2023.9)

S4 Bacteriome

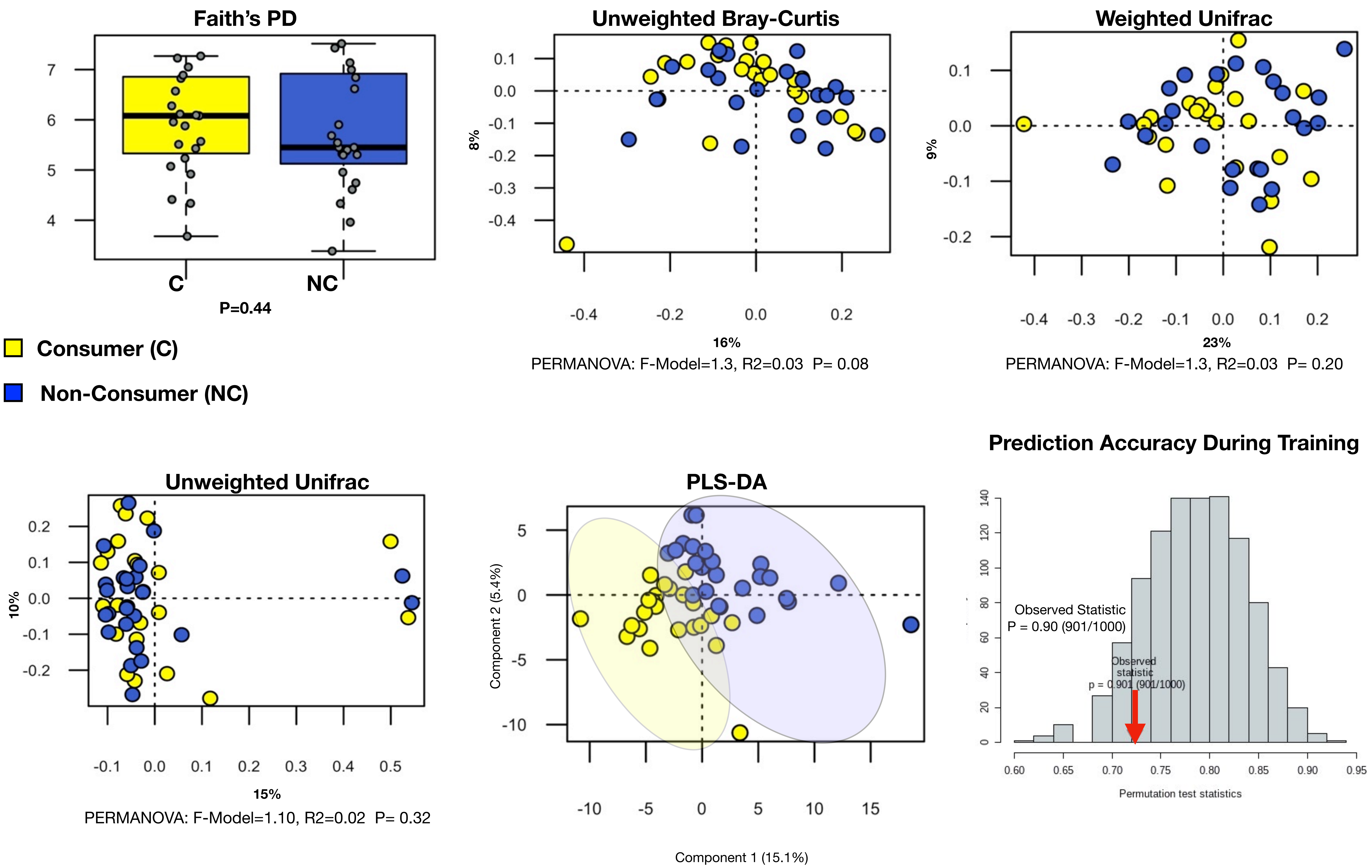

Supplement: Supplementary file 1 [file S2632289723000099sup001.zip › S2632289723000099sup004.pdf]
