# Supplementary material for: Regular consumption of lacto-fermented vegetables has greater effects on the gut metabolome compared with the microbiome
Source: Gut Microbiome (Camb). 2023 Jun 29;4:e11. doi: 10.1017/gmb.2023.9 (PMC11406409; doi:10.1017/gmb.2023.9)
Supplement: Supplementary file 1 [file S2632289723000099sup001.zip › S2632289723000099sup008.pdf]

S8 Power Calculation

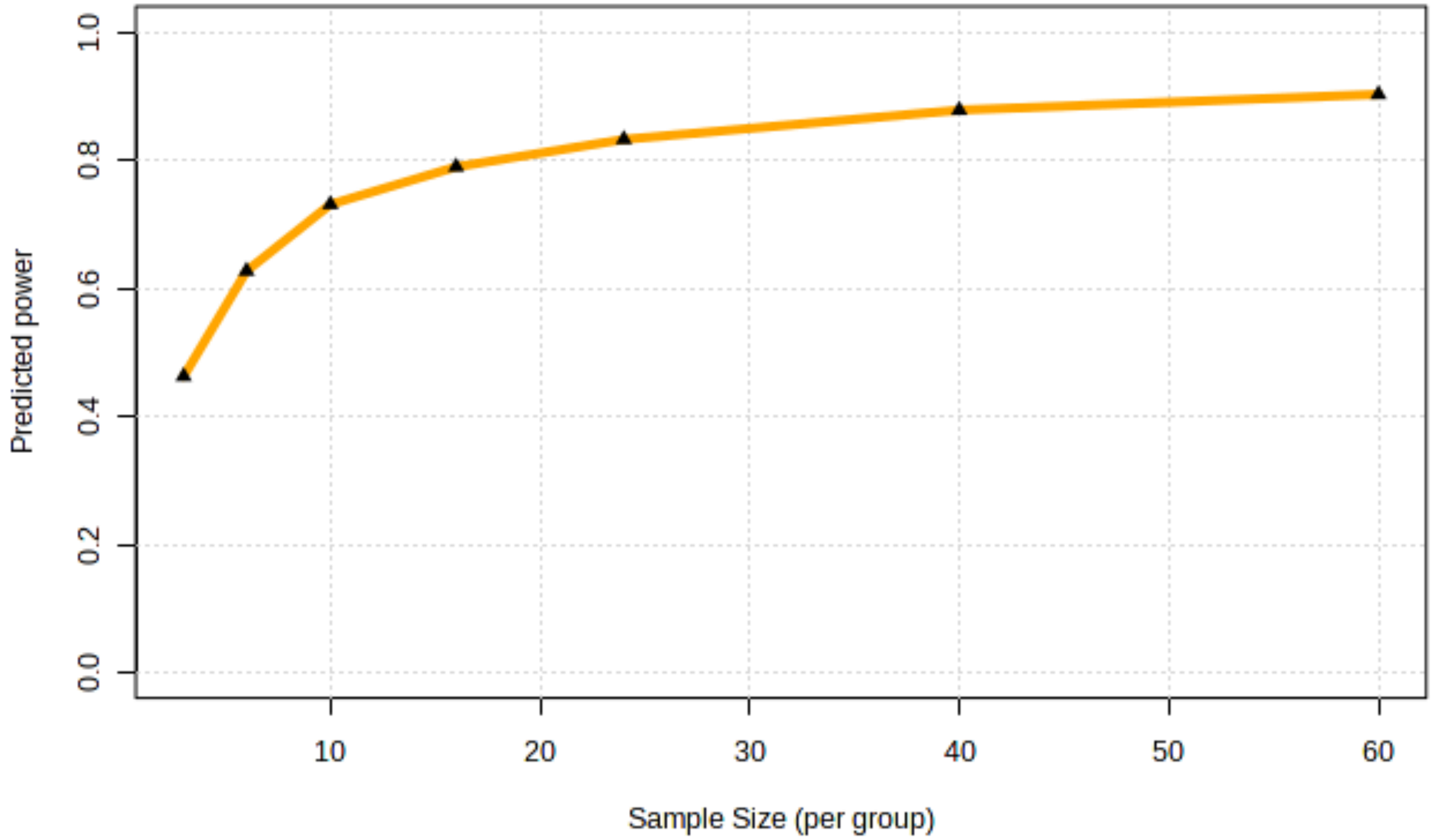

Power calculation based upon LC-MS data (calculated in Metaboanalyst 5.0). Sample size of 24; FDR=0.1.
