# Supplementary material for: Regular consumption of lacto-fermented vegetables has greater effects on the gut metabolome compared with the microbiome
Source: Gut Microbiome (Camb). 2023 Jun 29;4:e11. doi: 10.1017/gmb.2023.9 (PMC11406409; doi:10.1017/gmb.2023.9)
Supplement: Supplementary file 1 [file S2632289723000099sup001.zip › S2632289723000099sup011.docx]

HEI-2015 Dietary Components, Constituents and Scoring Standards

| Components | Units | Dietary Constituents | Maximum Score | **Standard for Maximum (*Standard for Minimum, if Moderation Component*)** |
| --- | --- | --- | --- | --- |
| **Total Fruits** | cup eq. | Total Fruit | 5 | ≥0.8 cup eq. per 1,000 kcal |
| **Whole Fruits** | cup eq. | Citrus, Melons, Berries + Other Intact Fruits | 5 | ≥0.4 cup eq. per 1,000 kcal |
| **Total Vegetables** | cup eq. | Total Vegetables + Legumes (Beans and Peas) in cup equivalents | 5 | ≥1.1 cup eq. per 1,000 kcal |
| **Greens and Beans** | cup eq. | Dark Green Vegetables + Legumes (Beans and Peas) in cup equivalents | 5 | ≥0.2 cup eq. per 1,000 kcal |
| **Whole Grains** | oz. eq. | Whole Grains | 10 | ≥1.5 oz eq. per 1,000 kcal |
| **Dairy** | cup eq. | Total Dairy | 10 | ≥1.3 cup eq. per 1,000 kcal |
| **Total Protein Foods** | oz. eq. | Total Meat, Poultry, and Seafood (including organ meats and cured meats) + Eggs + Nuts and Seeds + Soy + Legumes (Beans and Peas) in oz equivalents | 5 | ≥2.5 oz eq. per 1,000 kcal |
| **Seafood and Plant Proteins** | oz. eq. | Seafood (high in n-3) + Seafood (low in n-3) + Soy + Nuts and Seeds + Legumes (Beans and Peas) in oz equivalents | 5 | ≥0.8 oz eq. per 1,000 kcal |
| **Refined Grains** | oz. eq. | Refined Grains | 10 | ≤1.8 oz eq. per 1,000 kcal (*≥4.3 oz eq. per 1,000 kcal*) |
| **Added Sugars** | tsp. eq.^*^ | Added Sugars | 10 | ≤6.5% of energy (*≥26% of energy*) |
| **Fatty Acids** | g | (Total Monounsaturated Fatty Acids + Total Polyunsaturated Fatty Acids)/Total Saturated Fatty Acids | 10 | (MUFAs + PUFAs) /SFAs≥2.5 ((MUFAs + PUFAs)/SFAs≤1.2) |
| **Sodium** | mg^**^ | Sodium | 10 | ≤1.1 g per 1,000 kcal (*≥2.0 g per 1,000 kcal*) |
| **Saturated Fats** | g^***^ | Total Saturated Fatty Acids | 10 | ≤8% of energy (*≥16% of energy*) |

cup eq.=cup equivalents; oz. eq.=ounce equivalents; g=grams; mg=milligrams
*=teaspoon equivalents are converted to kcal in the scoring process.
**= sodium is converted from mg to g in scoring process.
***= fatty acids are calculated in grams but converted to energy in the scoring process.
