# Supplementary material for: Regular consumption of lacto-fermented vegetables has greater effects on the gut metabolome compared with the microbiome
Source: Gut Microbiome (Camb). 2023 Jun 29;4:e11. doi: 10.1017/gmb.2023.9 (PMC11406409; doi:10.1017/gmb.2023.9)
Supplement: Supplementary file 1 [file S2632289723000099sup001.zip › S2632289723000099sup012.docx]

**Inclusion Criteria “Consumers**

- Healthy subjects 20 years of age, but not more than 50 years of age at the time of enrollment.
- Must be able to provide signed and dated informed consent.
- Healthy subjects willing and able to provide a fecal sample.
- Subjects consuming 1 serving (2 ounces) of lacto-fermented vegetables (krauts, kimchis, other lacto-fermented vegetables) at least 5x a week.
- Subjects with vital signs and other physiological measurements generally falling within a normal range.

**Inclusion Criteria “Non-Consumers”**

- Healthy subjects 20 years of age, but not more than 50 years of age at the time of enrollment.
- Must be able to provide signed and dated informed consent.
- Healthy subjects willing and able to provide a fecal sample.
- Subjects with vital signs and other physiological measurements generally falling within a normal range.

**Exclusion Criteria for All Subjects**

- Subjects regularly consuming supplemental probiotics.
- Subjects implementing any drastic dietary changes within the past month, or that are trying to lose weight.
- Body Mass Index greater than or equal to 35 or less than or equal to 18.
- Vital signs outside of acceptable range at time of sample collection, i.e., blood pressure >160/100, oral temperature >100°F, pulse >100.
- Use of any of the following drugs within the last 6 months:
  - systemic antibiotics, antifungals, antivirals or antiparasitics (intravenous, I ntramuscular, or oral);
  - oral, intravenous, intramuscular, nasal or inhaled corticosteroids; cytokines;
  - methotrexate or immunosuppressive cytotoxic agents;
  - large doses of commercial probiotics consumed (greater than or equal to 10^8^ cfu or organisms per day) - includes tablets, capsules, lozenges, chewing gum or powders in which probiotic is a primary component.
  - Ordinary dietary components such as fermented beverages/milks, yogurts, foods do not apply, meaning they can be consumed. However, all subjects will be asked to limit other fermented food consumption at least two weeks before taking stool sample.
- For female subjects, combination hormone vaginal ring for contraception (due to unknown duration of local hormone effects).
- Acute disease at the time of enrollment (defer sampling until subject recovers). Acute disease is defined as the presence of a moderate or severe illness with or without fever.
- Chronic, clinically significant (unresolved, requiring on-going medical management or medication) pulmonary, cardiovascular, gastrointestinal, hepatic or renal functional abnormality, as determined by medical history or physical examination.
- History of cancer except for squamous or basal cell carcinomas of the skin that have been medically managed by local excision.
- Unstable dietary history as defined by major changes in diet during the previous month, where the subject has eliminated or significantly increased a major food group in the diet.
- Recent history of chronic alcohol consumption defined as more than five 1.5-ounce servings of 80 proof distilled spirits, five 12-ounce servings of beer or five 5-ounce servings of wine per day.
- Positive test for HIV, HBV or HCV.
- Any confirmed or suspected condition/state of immunosuppression or immunodeficiency (primary or acquired) including HIV infection.
- Major surgery of the GI tract, with the exception of cholecystectomy and appendectomy, in the past five years. Any major bowel resection at any time.
- History of active uncontrolled gastrointestinal disorders or diseases including:
  - inflammatory bowel disease (IBD) including ulcerative colitis (mild-moderate-severe), Crohn's disease (mild-moderate-severe), or indeterminate colitis;
  - irritable bowel syndrome (IBS) (moderate-severe);
  - persistent, infectious gastroenteritis, colitis or gastritis, persistent or chronic diarrhea of unknown etiology, Clostridium difficile infection (recurrent) or Helicobacter pylori infection (untreated);chronic constipation.
- Female who is pregnant or lactating.
- Treatment for or suspicion of ever having had toxic shock syndrome.
