# Supplementary material for: Regular consumption of lacto-fermented vegetables has greater effects on the gut metabolome compared with the microbiome
Source: Gut Microbiome (Camb). 2023 Jun 29;4:e11. doi: 10.1017/gmb.2023.9 (PMC11406409; doi:10.1017/gmb.2023.9)
Supplement: Supplementary file 1 [file S2632289723000099sup001.zip › S2632289723000099sup013.docx]

| Group | Sex | Age | Race/Ethnicity | Daily Avg. LFV Consumption (ounces) | HEI-2015 Index Score |
| --- | --- | --- | --- | --- | --- |
| LFV Consumer | Female | 32 | White/Caucasian | 4 | 33 |
| LFV Consumer | Female | unknown | White/Caucasian | 1 | 67 |
| LFV Consumer | Female | 34 | White/Caucasian and Hispanic | 3.8 | 84 |
| LFV Consumer | Male | 37 | White/Caucasian | 5.6 | 60 |
| LFV Consumer | Female | 37 | White/Caucasian | 5.6 | 66 |
| LFV Consumer | Female | 37 | White/Caucasian | 5.3 | 46 |
| LFV Consumer | Female | unknown | White/Caucasian | 9.3 | 60 |
| LFV Consumer | Female | unknown | White/Caucasian | .3 | 62 |
| LFV Consumer | Female | unknown | White/Caucasian | 2.6 | 65 |
| LFV Consumer | Female | 31 | White/Caucasian | 6 | 55 |
| LFV Consumer | Female | 28 | White/Caucasian | 8.3 | 50 |
| LFV Consumer | Male | 23 | Asian | 8.6 | 64 |
| LFV Consumer | Female | 33 | White/Caucasian | 4 | 68 |
| LFV Consumer | Female | 31 | Asian | 9 | 67 |
| LFV Consumer | Female | 30 | White/Caucasian | 3.5 | 53 |
| LFV Consumer | Female | 27 | White/Caucasian | 2 | 64 |
| LFV Consumer | Male | 30 | White/Asian | 5.3 | 54 |
| LFV Consumer | Male | 32 | White/Caucasian | 2.4 | 56 |
| LFV Consumer | Female | 31 | White/Caucasian | 4.3 | 62 |
| LFV Consumer | Female | 30 | White/Caucasian | 4 | 40 |
| LFV Consumer | Female | 33 | White/Caucasian | 2.5 | 58 |
| LFV Consumer | Female | 39 | White/Caucasian | 4 | 66 |
| LFV Consumer | Female | 43 | White/Caucasian | 2.6 | 61 |

Table 3 – Lacto-fermented vegetable (LFV) Consumers

*82.6% Female; 17.4% Male

*Average age 33.4

*82.6% White/Caucasian; 8.7% Asian; 4.3% White/Hispanic; 4.3% White Asian

*Average daily LFV intake: 4.5 ounces

*Average HEI-2015 Score: 59

Table 4 – Non-consumers

| Group | Sex | Age | Race/Ethnicity | HEI-2015 Index Score |
| --- | --- | --- | --- | --- |
| Non-Consumer | Female | unknown | White/Caucasian | 81 |
| Non-Consumer | Male | 23 | White/Caucasian | 24 |
| Non-Consumer | Male | unknown | Hispanic | --- |
| Non-Consumer | Female | 34 | White/Caucasian | 71 |
| Non-Consumer | Female | 37 | White/Caucasian | 56 |
| Non-Consumer | Male | unknown | Asian | 69 |
| Non-Consumer | Male | 26 | White/Caucasian | 48 |
| Non-Consumer | Female | 38 | White/Caucasian | 43 |
| Non-Consumer | Male | unknown | White/Caucasian | 54 |
| Non-Consumer | Female | unknown | White/Caucasian | 64 |
| Non-Consumer | Male | 31 | White/Caucasian | 56 |
| Non-Consumer | Female | 28 | White/Caucasian | 69 |
| Non-Consumer | Male | unknown | White/Caucasian | --- |
| Non-Consumer | Female | 24 | White/Caucasian | 56 |
| Non-Consumer | Female | 50 | White/Caucasian | 44 |
| Non-Consumer | Male | 28 | White/Caucasian | 31 |
| Non-Consumer | Female | 24 | White/Caucasian | 53 |
| Non-Consumer | Male | unknown | Asian | 64 |
| Non-Consumer | Female | 23 | White/Caucasian | 55 |
| Non-Consumer | Female | 47 | White/Caucasian | 49 |
| Non-Consumer | Male | 20 | White/Caucasian | 62 |
| Non-Consumer | Female | 21 | White/Caucasian | 39 |
| Non-Consumer | Female | 20 | White/Caucasian | 79 |
| Non-Consumer | Female | 20 | White/Caucasian | 38 |

*58.3% Female; 41.7% Male

*Average Age 29

*87.5% White/Caucasian; 8.3% Asian; 4.2% Hispanic

*Average HEI-2015 Score: 55
