# Supplementary figures and images for: Regular consumption of lacto-fermented vegetables has greater effects on the gut metabolome compared with the microbiome
Source: Gut Microbiome (Camb). 2023 Jun 29;4:e11. doi: 10.1017/gmb.2023.9 (PMC11406409; doi:10.1017/gmb.2023.9)

Figure S1 Flow Chart

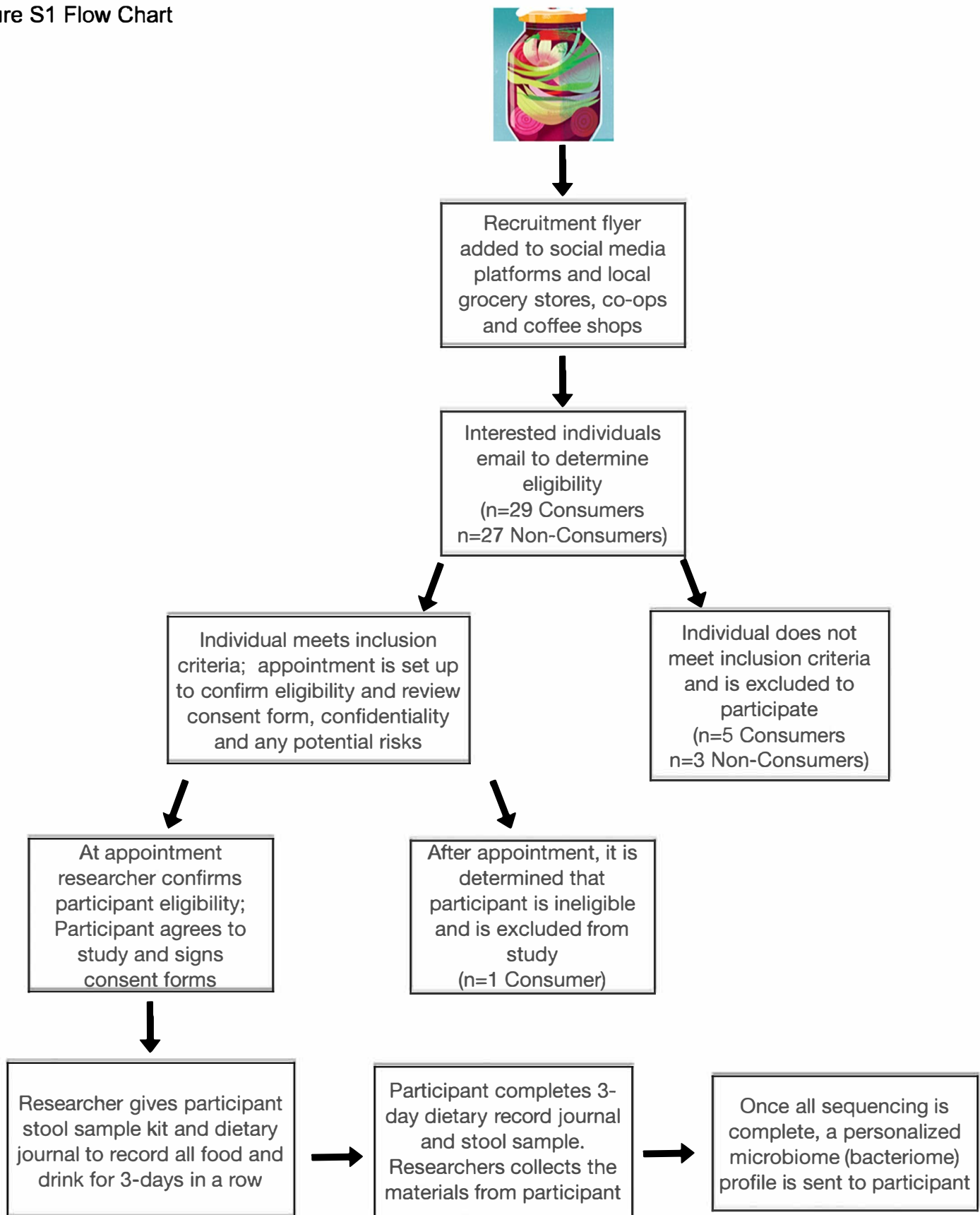

Supplement: Supplementary file 1 [file S2632289723000099sup001.zip › S2632289723000099sup001.pdf]

# S2 Dietary Intake

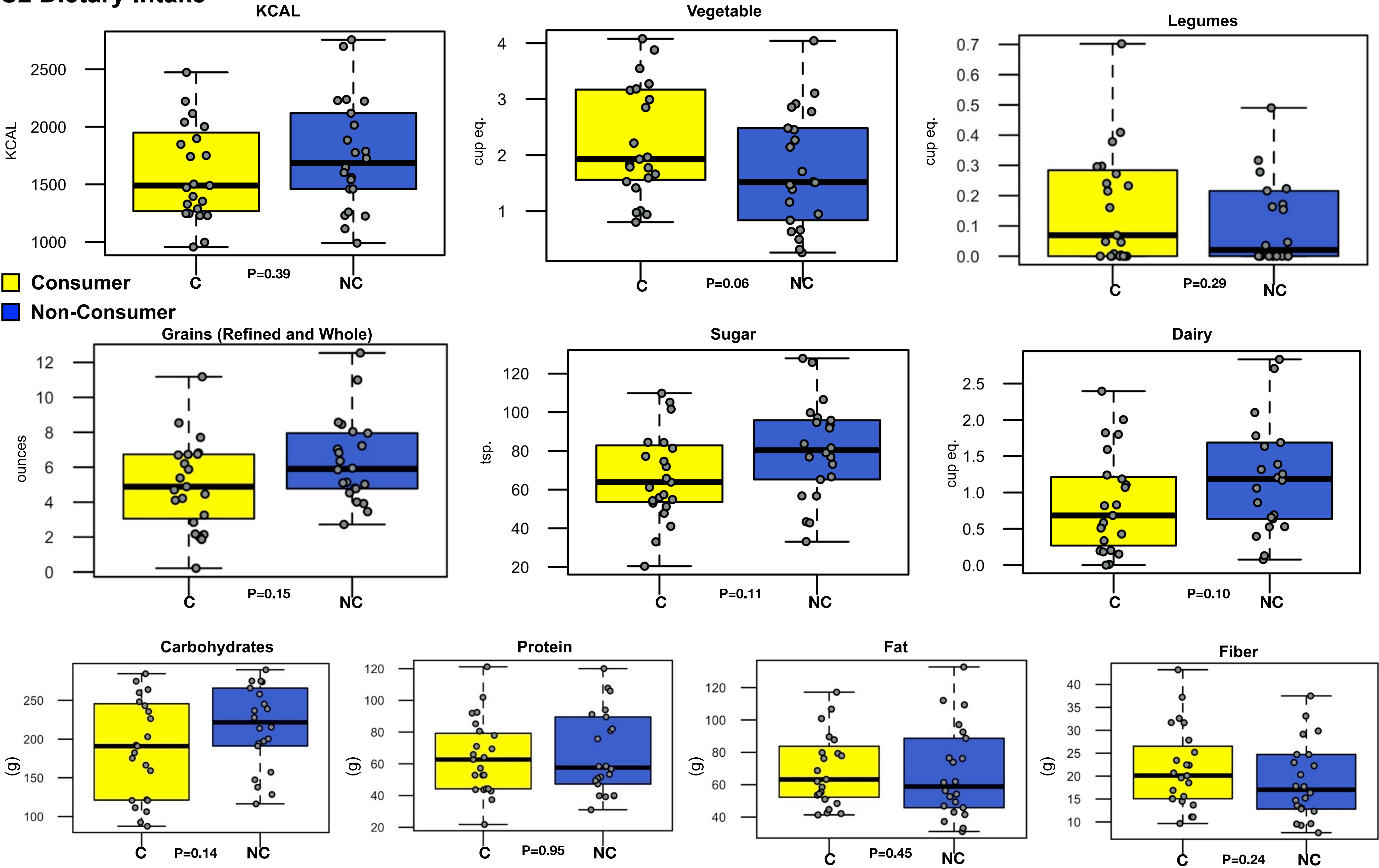

Supplement: Supplementary file 1 [file S2632289723000099sup001.zip › S2632289723000099sup002.pdf]

Healthy Eating Index 2015 - Dietary Components of LFV Consumers and Non-Consumers

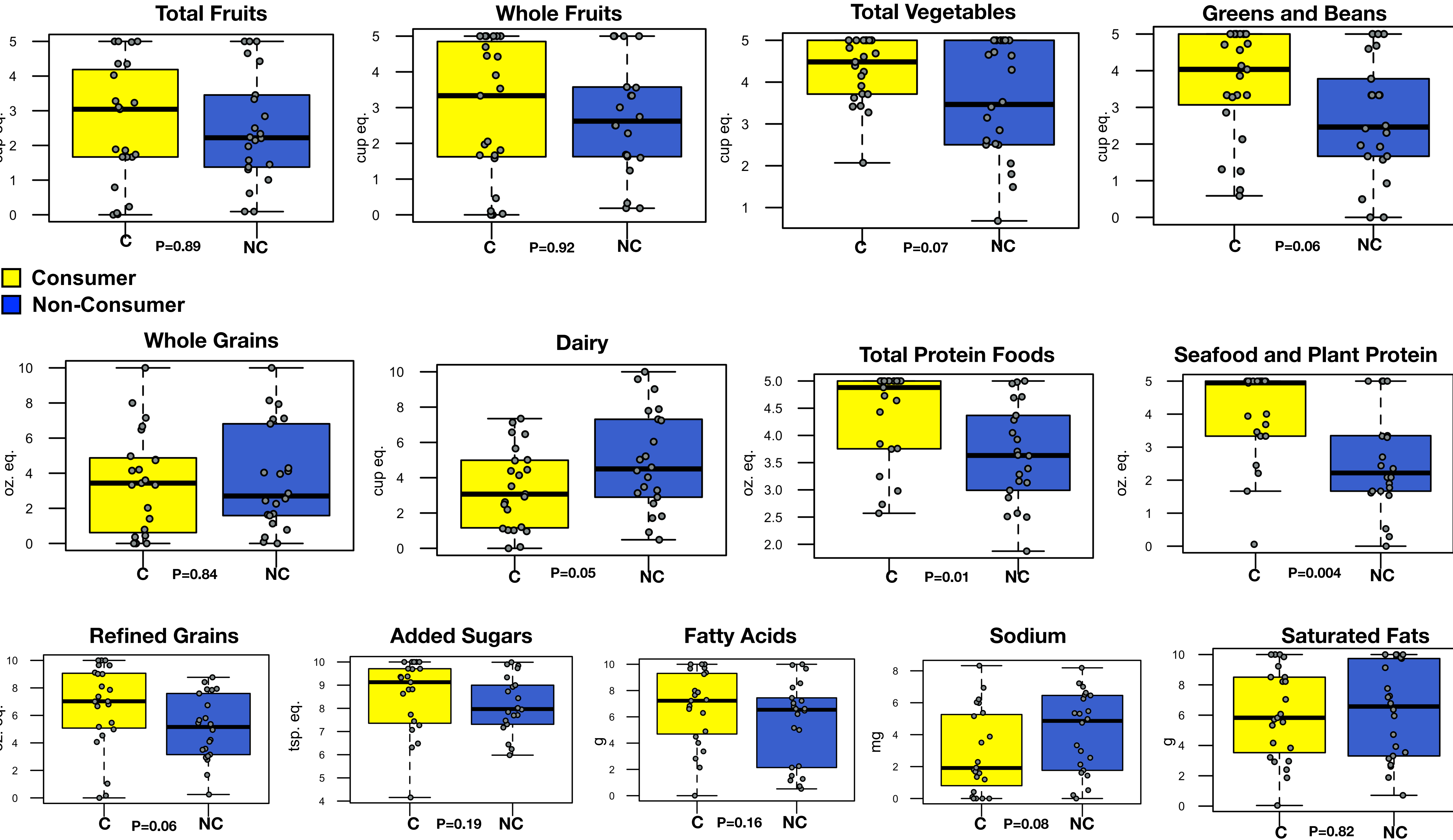

Supplement: Supplementary file 1 [file S2632289723000099sup001.zip › S2632289723000099sup003.pdf]

S5    **Boxplots of Discriminating Bacterial Taxa**

**Consumer (C)**

**Non-Consumer (NC)**

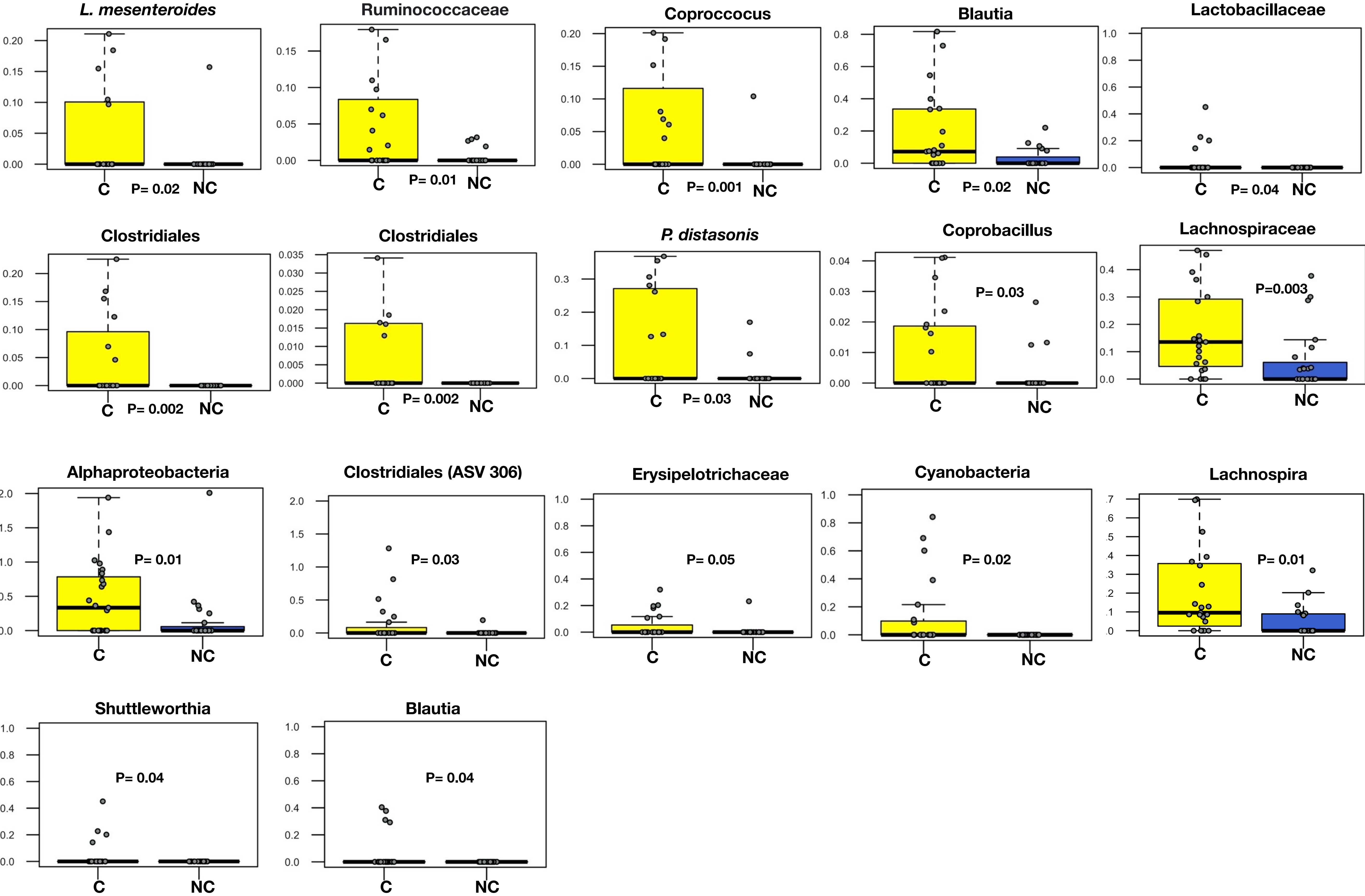

Supplement: Supplementary file 1 [file S2632289723000099sup001.zip › S2632289723000099sup005.pdf]

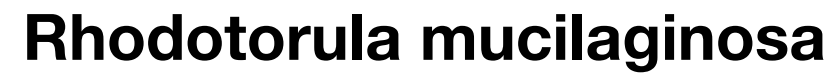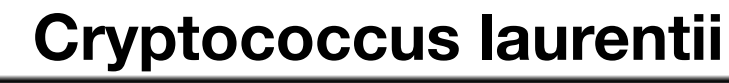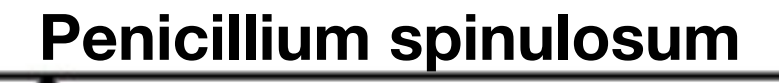

## Consumer (C)



## Non-Consumer (NC)

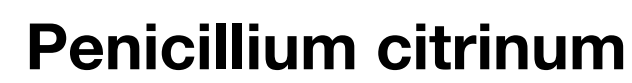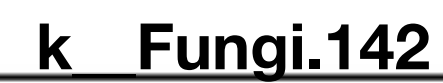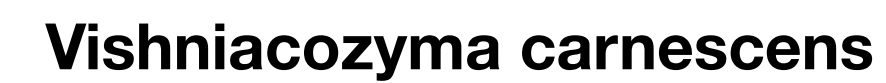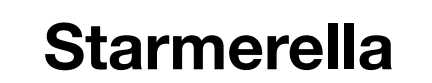

Supplement: Supplementary file 1 [file S2632289723000099sup001.zip › S2632289723000099sup007.pdf]

# S9 Principal Component Analysis

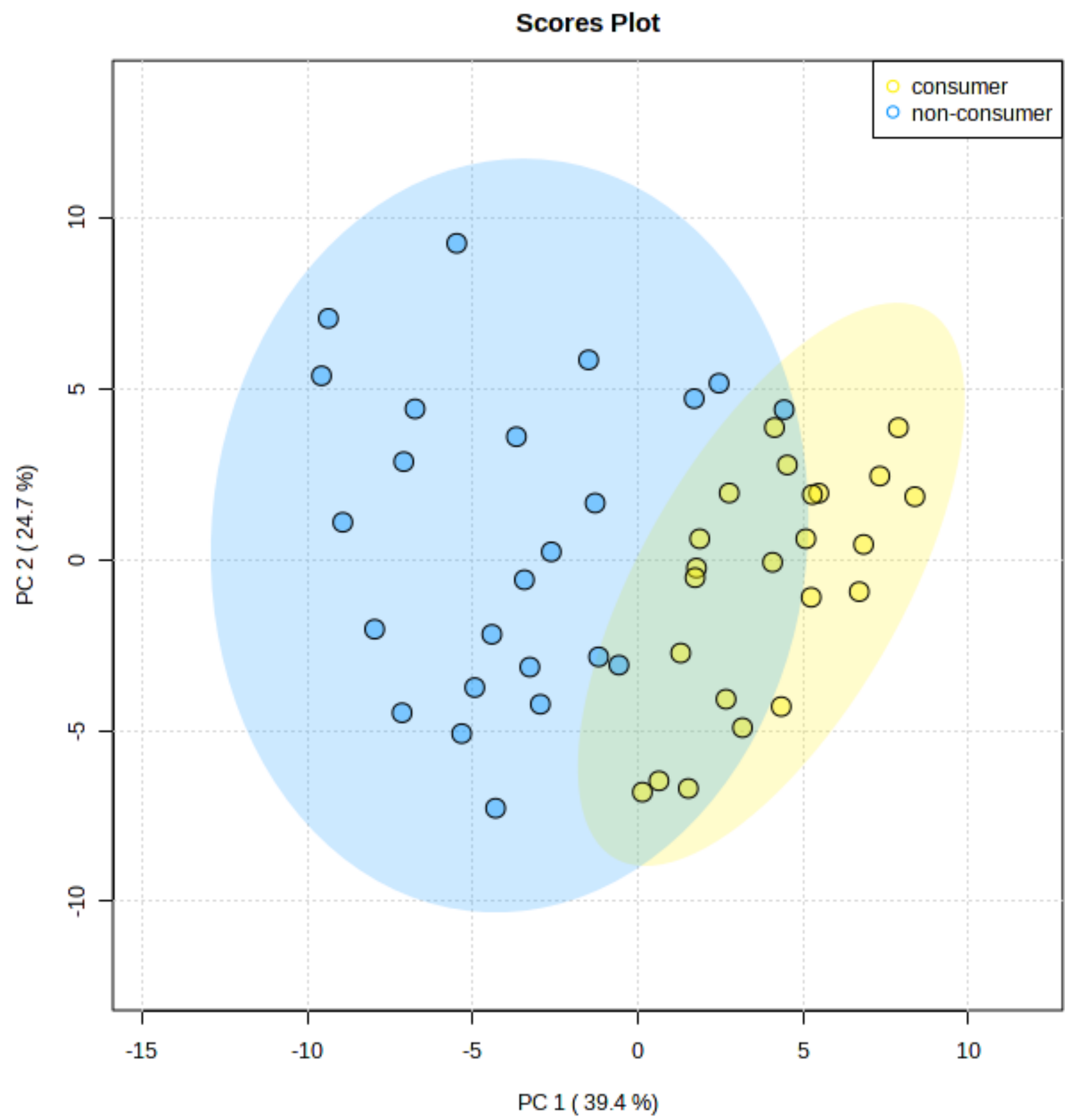

Supplement: Supplementary file 1 [file S2632289723000099sup001.zip › S2632289723000099sup009.pdf]

S10 Boxplots of Discriminant Metabolites

Consumer (C)  
Non-Consumer (NC)

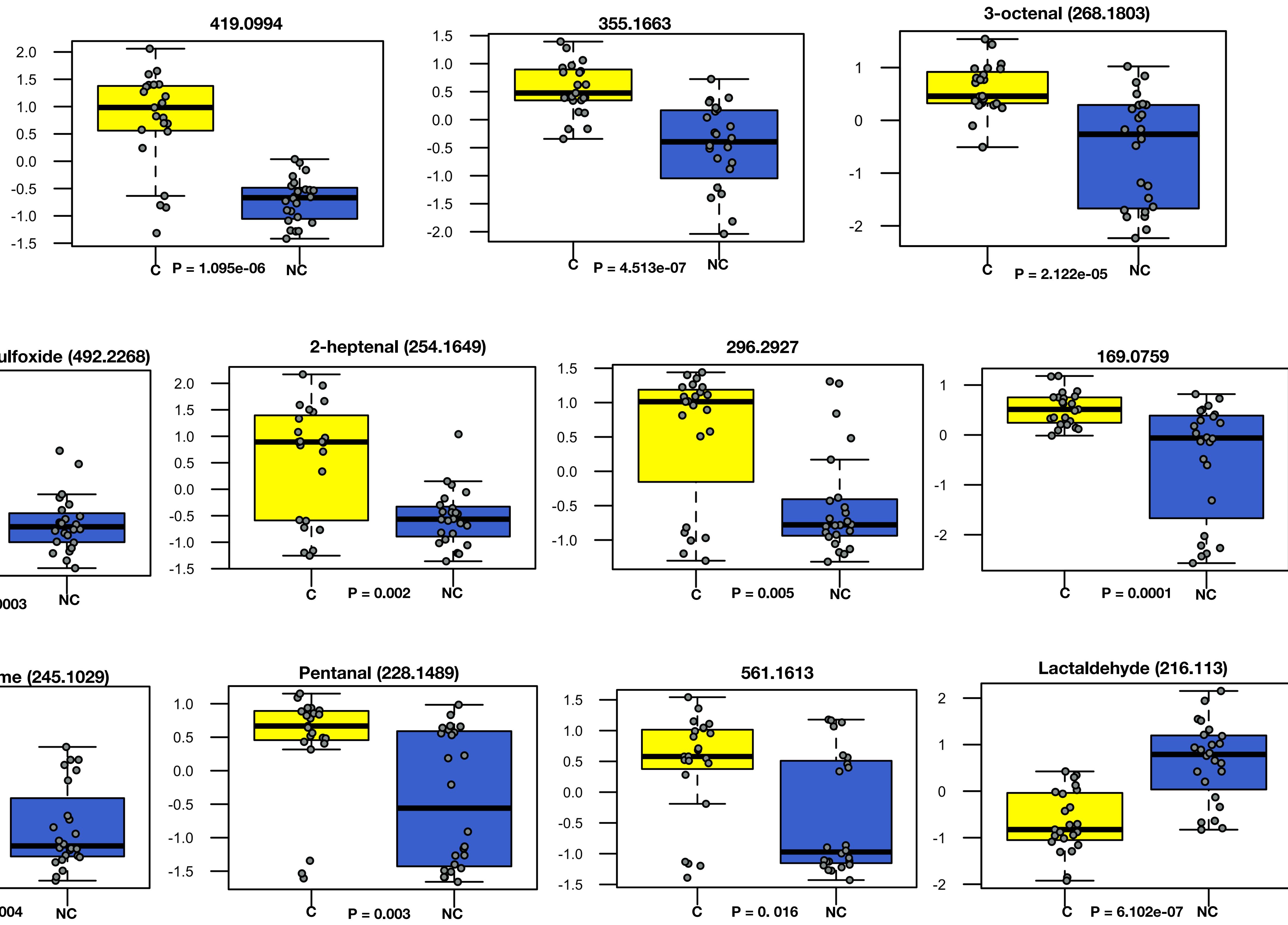

Supplement: Supplementary file 1 [file S2632289723000099sup001.zip › S2632289723000099sup010.pdf]
